# 定量磷酸化蛋白质组解析17*β*-雌二醇致死效应的细胞调控过程

**DOI:** 10.3724/SP.J.1123.2023.04025

**Published:** 2024-04-08

**Authors:** Yanan LI, Xiaoyan LIU, Yan WANG, Zhen LIU, Mingliang YE, Hailin WANG

**Affiliations:** 1.国科大杭州高等研究院环境学院, 浙江 杭州 310024; 1. School of Environment, Hangzhou Institute for Advanced Study, University of Chinese Academy of Sciences, Hangzhou 310024, China; 2.中国科学院生态环境研究中心, 环境化学与生态毒理学国家重点实验室, 北京 100085; 2. State Key Laboratory of Environmental Chemistry and Eco-toxicology, Research Center for Eco-Environmental Sciences, Chinese Academy of Sciences, Beijing 100085, China; 3.中国科学院大连化学物理研究所, 中国科学院分离分析化学重点实验室, 辽宁 大连 116023; 3. CAS Key Laboratory of Separation Science for Analytical Chemistry, Dalian Institute of Chemical Physics, Chinese Academy of Science, Dalian 116023, China

**Keywords:** 液相色谱-串联质谱, 固定化钛离子亲和色谱, 数据非依赖采集, 磷酸化蛋白质组, 17*β*-雌二醇, 雌激素, 致死效应, liquid chromatography-tandem mass spectrometry (LC-MS/MS), immobilized titanium ion affinity chromatography (Ti^4+^-IMAC), data-independent acquisition (DIA), phosphoprotoemics, 17*β*-estradiol (E2), estrogen, lethal effect

## Abstract

17*β*-雌二醇(E2)是人体内一种重要的内分泌激素,在生理浓度下(0.2~1.0 nmol/L)对生殖系统、乳腺等靶器官的生长发育起着重要的调节作用。但很多研究表明,高剂量(μmol/L~mmol/L)的E2能够诱导肿瘤组织消退和细胞凋亡,其具体调控机制尚不明确。本工作聚焦于高剂量(μmol/L)的E2致死效应,首先分析了μmol/L水平的E2对HeLa细胞表型的影响,发现在1~10 μmol/L下E2以浓度依赖的形式抑制HeLa细胞增殖,并诱导HeLa细胞发生死亡,其中,用5 μmol/L E2处理2天后可使约74%的HeLa细胞增殖受到抑制,并引起约50%的HeLa细胞死亡。在此基础上,为了探究高剂量E2诱导细胞死亡的内在调控过程,将基于固相萃取(SPE)的固定化钛离子亲和色谱技术(Ti^4+^-IMAC)与基于数据非依赖采集模式(DIA)的蛋白质组定量技术结合,用于筛选HeLa细胞内参与高剂量(μmol/L)E2致死效应调控过程的磷酸化位点。最终,在5 μmol/L E2和二甲基亚砜(DMSO)处理的HeLa细胞中共鉴定到超过10000个磷酸化位点; *t*检验分析发现, 在E2处理后, 有924个磷酸化位点(对应599个蛋白质)的丰度发生了显著变化(显著性水平(*p*)<0.01, |log_2_(倍数变化)|≥1), 推测其可能参与调控E2致死效应过程。此外,有453个磷酸化位点(对应325个蛋白质)仅单独发生在E2或DMSO处理后的HeLa细胞样品中,表明这些磷酸化位点在E2处理后发生了磷酸化或去磷酸化,也可能参与E2致死效应的调控过程。分别对以上两种方式筛选的E2调控的磷酸化蛋白质进行富集分析, 发现这些磷酸化蛋白质主要参与细胞分裂、核糖体/核质转运、信使核糖核酸(mRNA)加工/剪接及转录等过程,表明高剂量的E2可能通过调控核糖体及mRNA加工等过程影响蛋白质转录,进而诱导细胞发生死亡。此外,我们发现表皮生长因子受体(EGFR)和丝裂原活化蛋白激酶(MAPK)家族蛋白(包括MAPK1、MAPK4和MAPK14)上多个磷酸化位点的修饰水平在高剂量E2处理后发生了明显变化,表明EGFR和MAPK信号通路可能在雌激素诱导的细胞死亡中起着重要调控作用。本实验得到的磷酸化蛋白质组定量结果有助于进一步了解高剂量E2的内在调控过程,为后续解析高剂量E2的作用机制及疾病的治疗提供了参考。

## Abstract (en)

17*β*-Estradiol (E2), an important endocrine hormone in the mammalian body, participates in the regulation of the physiological functions of the reproductive system, mammary glands, bone, and cardiovascular system, among others. Paradoxically, despite the physiological actions of endogenous E2 (0.2-1.0 nmol/L), numerous clinical and experimental studies have demonstrated that high-dose E2 treatment can cause tumor regression and exert pro-apoptotic actions in multiple cell types; however, the underlying mechanism remains undescribed. In particular, little information of the cellular processes responding to the lethality of E2 is available.

In the present study, we attempted to characterize the cellular processes responding to high-dose (μmol/L) E2 treatment using quantitative phosphoproteomics to obtain a better understanding of the regulatory mechanism of E2-induced cell death. First, the cell phenotype induced by high-dose E2 was determined by performing Cell Counting Kit-8 assay (CCK8), cell cytotoxicity analysis by trypan blue staining, and microscopic imaging on HeLa cells treated with 1-10 μmol/L E2 or dimethyl sulfoxide (DMSO) for 1-3 d. E2 inhibited cell proliferation and induced cell death in a dose- and time-dependent manner. Compared with the DMSO-treated HeLa cells, the cells treated with 5 μmol/L E2 for 2 d demonstrated >74% growth inhibition and approximately 50% cell death. Thus, these cells were used for quantitative phosphoproteomic analysis. Next, a solid-phase extraction (SPE)-based immobilized titanium ion affinity chromatography (Ti^4+^-IMAC) phosphopeptide-enrichment method coupled with data-independent acquisition (DIA)-based quantitative proteomics was employed for the in-depth screening of high-dose E2-regulated phosphorylation sites to investigate the intracellular processes responding to high-dose E2 treatment. Liquid chromatography-tandem mass spectrometry (LC-MS/MS) identified over 10000 phosphorylation sites regulated by E2 and DMSO in HeLa cells. In comparison with the DMSO-treated cells, the cells treated with 5 μmol/L E2 showed 537 upregulated phosphorylation sites and 387 downregulated phosphorylation sites, with a threshold of *p*<0.01 and |log_2_(fold change)|≥1. A total of 924 phosphorylation sites on 599 proteins were significantly regulated by high-dose E2, and these sites were subjected to enrichment analysis. In addition, 453 differently regulated phosphorylation sites on 325 proteins were identified only in the E2- or DMSO-treated cell samples. These phosphorylation sites may be phosphorylated or dephosphorylated in response to high-dose E2 stimulation and were subjected to parallel enrichment analyses. Taken together, 1218 phosphorylation sites on 741 proteins were significantly regulated by high-dose E2 treatment. The functional phosphoproteins in these two groups were then analyzed using Gene Ontology (GO) and Gene Set Enrichment Analysis (GSEA) to determine the biological processes in which they participate and the Kyoto Encyclopedia of Genes and Genomes (KEGG) pathway database. Consistent with the cell-phenotype data, cell cycle-related proteins were highly enriched in the two groups of E2-regulated phosphoproteins (*p*<0.05), indicating that high-dose E2 treatment can regulate cell proliferation. In addition, E2-regulated phosphoproteins were highly enriched in the cellular processes of ribosome biogenesis, nucleocytoplasmic transport, and messenger ribonucleic acid (mRNA) processing/splicing (*p*<0.05), indicating that the activation of these processes may contribute to high-dose E2-induced cell death. These results further confirm that high-dose E2 treatment inhibits protein translation and induces cell death. Furthermore, the significant upregulation of multiple phosphorylation sites associated with epidermal growth factor receptor (EGFR) and mitogen-activated protein kinases (MAPKs) MAPK1, MAPK4, and MAPK14 by high-dose E2 indicates that the EGFR and MAPK signaling pathways are likely involved in the regulation of E2-induced cell death. These phosphorylation sites likely play vital roles in E2-induced cell death in HeLa cells. Overall, our phosphoproteomic data could be a valuable resource for uncovering the regulatory mechanisms of E2 in the micromolar range.

17*β*-雌二醇(17*β*-estradiol, E2)是人体内活性最强的内源性激素,主要在卵巢中合成,通过人体循环作用于靶器官,在许多生理和病理功能的调节中起着至关重要的作用^[1]^。血浆中的雌激素浓度通常在0.2~1.0 nmol/L内,但在胎盘、脑等产生激素的局部环境中,E2的浓度及活性会远高于循环内水平,甚至高达12 μmol/L^[2]^。机体内细胞对雌激素的反应主要由雌激素受体(estrogen receptor, ER)*α*和*β*介导,二者结合后以直接或间接的方式结合到靶基因雌激素调控元件(estrogen regulating elements, ERE)上,进而调控靶基因转录,促进或抑制相关靶基因表达,调控机体生长发育等重要生理、病理过程^[1,3,4]^。已有研究证实,乳腺癌、骨质疏松症、心血管及子宫内膜癌等疾病均与雌激素失衡有关^[5]^。同时,临床上的卵巢切除术、激素替代治疗(HRT)等方式可通过调控机体内雌激素的水平用于癌症、雌激素缺乏等相关疾病的治疗^[6]^。值得注意的是,除了促生长效应,许多临床和实验研究显示E2能够促进肿瘤组织消退,诱导多种细胞发生死亡,呈现复杂的非单调剂量-效应响应关系^[7⇓⇓-10]^。因此,阐明雌激素的内在调控过程,可能对雌激素作用机制的理解及相关临床治疗方案的制定有一定帮助。但遗憾的是,目前大部分研究主要关注E2在低剂量(pmol/L~nmol/L)下的作用机制及生理功能,对于高剂量(μmol/L)E2的相关研究则较少,其内在调控过程、作用机制及其生理、病理功能尚不明确^[11]^。2019年王晓东课题组^[2]^发现,μmol/L水平的E2可以诱导HeLa细胞发生凋亡;接着通过全基因筛选及免疫共沉淀技术发现,E2与PDE3A(cGMP-inhibited 3',5'-cyclic phosphodiesterase 3A)直接结合,诱导PDE3A与SLFN12(Schlafen family member 12)相互作用形成E2-PDE3A-SLFN12复合物,破坏核糖体与信号识别颗粒之间的作用,抑制蛋白质翻译,促进宫颈癌HeLa细胞发生凋亡,并发现这一调控可能在人胎盘的侵染和发育过程中起到重要作用。但是,目前尚未有研究从蛋白质组学层次全面解析高剂量E2致死效应过程中的细胞内调控过程,这有助于进一步了解高剂量E2的作用机制,为相关疾病的治疗提供参考。

近年来,随着质谱技术的不断进步,单次实验可以实现成千上万个蛋白质及其翻译后修饰(posttranslational modifications, PTMs,包括磷酸化、泛素化等)的高灵敏鉴定和高精准定量分析。蛋白质组学(包括翻译后修饰等变体)也逐渐成为药物等分子作用机制研究的重要工具^[12⇓⇓-15]^。蛋白质磷酸化是最重要也是研究最广泛的蛋白质翻译后修饰之一,它可以协调细胞内多种生物进程的发生与发展^[16,17]^。生物样品具有高度复杂、磷酸化蛋白质丰度低及高度动态变化等特性,对磷酸化蛋白质组学样品进行高特异性富集是降低样品复杂度、提高磷酸化蛋白质及其位点鉴定覆盖度至关重要的一步。目前,固定化金属离子亲和色谱(IMAC, Ti^4+^、Fe^3+^、Ga^3+^、Zr^4+^等)和金属氧化物亲和色谱(MOAC)是两种主要的磷酸化肽段富集手段,其中我们课题组^[18]^发展的基于Ti^4+^-IMAC单分散微球的富集方法在磷酸化蛋白质组学中得到了广泛的应用。此外,与传统的基于数据依赖采集(data-dependent acquisition, DDA)的鸟枪蛋白质组学相比,基于数据非依赖采集模式(data-independent acquisition, DIA)的蛋白质组定量技术可通过质谱连续获取在一定隔离窗口宽度内全部母离子产生的碎片离子,进一步提升蛋白质鉴定的灵敏度和定量准确性,近年来在蛋白质组学领域得到越来越多的关注^[19⇓-21]^。

基于此,为了探究高剂量E2致死效应的内在调控过程,首先分析了μmol/L水平的E2对HeLa细胞表型的影响,并建立了高剂量E2致死HeLa细胞模型;接着将基于Ti^4+^-IMAC的磷酸肽富集方法与基于DIA模式的蛋白质组定量方法结合,用于HeLa细胞中参与调控高剂量E2致死效应的磷酸化位点的全面筛选,并基于现有蛋白质功能数据库的注释信息,初步探究了这些磷酸化位点可能参与调控的细胞过程,为后续高剂量E2作用机制的阐明提供一定的数据支持。

## 1 实验部分

### 1.1 仪器、试剂与材料

装配有UltiMate 3000 RSLC纳升级液相色谱系统的Q Exactive HF质谱仪、Countess全自动细胞计数仪(美国Thermo Fisher Scientific公司);JY92-Ⅱ超声破碎仪(宁波新芝生物科技股份有限公司)。

盐酸胍(GdmCl,纯度≥99%)、三(2-羧乙基)膦盐酸盐(TCEP,纯度≥95%)、2-氯乙酰胺(CAA,纯度≥98%)、氯化钠(NaCl,纯度≥99%)、氟化钠(NaF,纯度≥99%)、钒酸钠(Na_3_VO_4_,纯度≥90%)、焦磷酸钠(Na_4_O_7_P_2_,纯度≥95%)、*β*-甘油磷酸钠(Na_2_C_3_H_7_O_6_P,纯度≥99%)、氨水(质量分数28%)、碳酸氢铵(NH_4_HCO_3_,纯度≥99.5%)、4-羟乙基哌嗪乙磺酸(HEPES,纯度≥99%)、蛋白酶抑制剂Cocktail、三氟乙酸(TFA,纯度≥99%)、甲酸(FA,纯度>99%)、尿素(纯度≥98%)、E2(纯度≥98%)、二甲基亚砜(DMSO,纯度≥99.9%)等试剂均购自美国Sigma-Aldrich公司;测序级胰蛋白酶购自北京华利世科技有限公司;Countess^TM^细胞计数腔室载玻片及台盼蓝染色剂购自美国Thermo Fisher Scientific公司;Cell Counting Kit-8(CCK-8)试剂盒、青霉素-链霉素储存液和二喹啉甲酸法(BCA)试剂盒均购自上海碧云天生物技术有限公司;三羟甲基氨基甲烷(Tris,纯度≥99%)购自生工生物工程(上海)股份有限公司;iRT(indexed retention time)标准肽段混合物购自瑞士Biognosys公司;RPMI 1640培养基、磷酸缓冲盐溶液(PBS)和小牛血清(BS)购自美国Gibco公司;细胞培养皿购自美国Corning公司;乙腈(ACN, HPLC级)、甲醇(MeOH, HPLC级)、异丙醇(IPA, HPLC级)、超纯水(质谱级)购自德国Merck公司。

Sep-Pak C18固相萃取柱购自美国Waters公司;色谱填料1.9 μm C18-AQ购自德国Dr. Maisch公司;熔融石英毛细管购自美国Polymicro Technologies公司;除液相色谱-串联质谱系统的流动相外,其他实验溶液所用的超纯水均由Milli-Q系统(Millipore,美国)制备;10 kDa超滤离心管购自德国Sartorius公司。

### 1.2 细胞培养与计数

细胞培养:将HeLa细胞复苏于含有10% BS、100 U/mL青霉素和100 U/mL链霉素的RPMI 1640培养基(完全培养基)中,并在37 ℃、5% CO_2_气体浓度、湿度适宜的恒温培养箱中进行培养。

细胞计数:将HeLa细胞接种于六孔板中,培养24 h,用PBS清洗后加入新鲜的完全培养基,实验组中加入E2(储备液为500 μmol/L,溶解溶剂为DMSO)至终浓度为5 μmol/L,对照组中加入等体积的DMSO;分别在E2/DMSO处理前和处理后的第1、2、3天进行显微镜拍照,记录细胞形态,并用胰蛋白酶消化后重悬;取等体积的单细胞悬液与等体积的台盼蓝进行充分混合,转移至Countess^TM^细胞计数腔室载玻片,使用全自动细胞计数仪进行细胞计数,记录活细胞和死细胞的数量及比例。每种处理进行3个复孔分析,每个样品重复计数3次。

### 1.3 细胞增殖与毒性实验

将HeLa细胞接种至96孔板,培养24 h后加入空白完全培养基和含有一定浓度E2/DMSO的完全培养基至特定时间(分别设置为对照组和实验组),加入10 μL CCK-8试剂,在37 ℃下孵育2 h后,在450 nm波长处测定吸光度值;每种处理进行6个复孔分析,每个CCK-8试剂处理时间点(E2/DMSO处理前和处理后的第1、2、3天)设置一个溶剂对照组,通过(*A*_实验组_-*A*_溶剂对照组_)/(*A*_对照组_-*A*_溶剂对照组_)来计算细胞存活率,得到的数值在GraphPad Prism软件^[22]^中进行后续数据处理。

### 1.4 细胞破碎和蛋白质提取

用预冷的PBS溶液重复清洗细胞3次后,弃去PBS溶液,倒入液氮,加入95 ℃预热、含有1%蛋白酶抑制剂混合物的细胞裂解液(6 mol/L GdmCl、10 mmol/L TCEP、40 mmol/L CAA、25 mmol/L Tris、1 mmol/L NaF、1 mmol/L Na_3_VO_4_、10 mmol/L Na_4_O_7_P_2_、1 mmol/L Na_2_C_3_H_7_O_6_P, pH 8.0),在冰浴上将细胞轻轻刮取于15 mL离心管中,之后将离心管置于95 ℃水浴中加热5 min,再置于冰浴中冷却15 min;随后使用探头式超声波破碎仪进行细胞破碎(输出功率为200 W, 20次循环,模式为3 s开/3 s关)。将破碎后的细胞裂解液置于95 ℃水浴中加热5 min进行还原烷基化后,于冰浴中冷却15 min,随后在4 ℃、4000 g条件下离心30 min,将上清液转移至新的离心管中并采用BCA(bicinchoninic acid)法测定蛋白质浓度。

### 1.5 蛋白质酶解

将上述还原烷基化后的蛋白质样品转移至500 μL 10 kDa超滤管中,进行基于滤膜辅助的样品制备流程(filter aided sample preparation, FASP)^[23]^。首先,在20 ℃、14000 g条件下离心,尽量去除超滤管中的溶液;加入100 μL 100 mmol/L HEPES溶液(pH 8.0),在20 ℃、14000 g条件下离心,重复两次;随后更换新的滤出液收集管,向滤膜中加入80 μL 50 mmol/L HEPES溶液,按照1∶30(胰蛋白酶∶蛋白质)的质量比加入胰蛋白酶,于37 ℃下酶解过夜;酶解完成后,于20 ℃、14000 g条件下离心20 min,收集滤出液;随后向滤膜中加入50 μL 10 mmol/L HEPES溶液,在14000 g下离心20 min,重复两次;合并收集的滤出液,与2 mol/L尿素等体积混合,待富集磷酸肽使用。

### 1.6 磷酸肽富集

用Ti^4+^-IMAC填充离心小柱的制备过程如下:将直径为1 mm的聚四氟乙烯筛板填充于200 μL枪头中并固定在距枪头尖端约0.5 cm的位置作为塞子,然后将分散于0.1% TFA水溶液中的Ti^4+^-IMAC微球转移到枪头中,得到Ti^4+^-IMAC填充的离心小柱;离心装置由两个离心管组成,一个600 μL的离心管用于固定离心小柱的尖端,另一个1.5 mL的离心管用于离心支撑和样品收集。

采用Ti^4+^-IMAC填充离心小柱富集磷酸化肽段的过程^[24]^如下:首先,以1∶20(肽段∶微球)的质量比取Ti^4+^-IMAC微球均分于自制的离心小柱内,加入200 μL 0.1% TFA水溶液清洗材料;接着,向上述酶解后的肽段溶液中加入TFA至其体积分数为1%,在室温、12000 g下离心5 min,转移上清液至新的离心管中,与上样缓冲液(ACN-TFA-H_2_O (80∶6∶14, v/v/v))等体积混合后,加入平衡后的Ti^4+^-IMAC微球进行富集,随后依次使用洗液1(含有200 mmol/L NaCl的ACN-TFA-H_2_O(50∶6∶44, v/v/v))洗涤3次,再用洗液2(ACN-TFA-H_2_O, 30∶0.1∶69.9, v/v/v)洗涤1次,每次使用200 μL;洗涤完成后,向离心小柱中加入10%的氨水进行洗脱,每次使用200 μL,洗脱两次,合并洗脱液,冷冻干燥后用1% TFA水溶液复溶,再用C18固相萃取柱进行除盐、冻干,得到富集好的磷酸肽,保存于-80 ℃备用。

### 1.7 液相色谱-串联质谱分析

液相色谱流动相A为0.1%甲酸水溶液,流动相B为含80%ACN的0.1%甲酸水溶液。将磷酸肽样品复溶在0.1%甲酸水溶液中,以9∶1(v/v)的比例加入iRT肽段溶液,混合后以5 μL/min的流速上样至装填有1.9 μm C18填料的预柱(3 cm×200 μm)中,分析时间为20 min。流动相梯度设置:0~10 min, 2%B; 10~11 min, 6%B; 11~20 min, 12%B。之后,将在预柱上富集到的样品以600 nL/min的流速上样至实验室自制的毛细管喷针一体分析柱(35 cm×150 μm, 1.9 μm C18)中,置于55 ℃柱温箱中进行反相色谱分离,分析时间为90 min。流动相梯度设置:0~58 min, 12%B~30%B; 58~70 min, 30%B~45%B; 70~73 min, 45%B~90%B; 73~81.5 min, 90%B; 81.5~82 min, 90%B~2%B; 82~90 min, 2%B。

Q Exactive HF质谱采用正离子、DIA模式:一级质谱扫描质荷比(*m/z*)为350~1050,均分为24个分段窗口,分辨率设置为60000(*m/z* 200),离子允许的最大注入时间为20 ms,允许注入最大电荷数为3×10^6^。将收集到的离子依据*m/z*依次进行扫描,归一化碰撞碎裂能量设置为27%,二级质谱分辨率设置为30000(*m/z* 200),离子注入时间设置为“auto”,允许注入最大电荷数为1×10^6^。

在E2和DMSO处理组中分别设置4个生物学重复样品,对每个生物学重复的细胞样品进行一次液相色谱-串联质谱分析。由质谱分析产生的Raw文件已上传至JPOST Repository,对应的JPOST和ProteomeXchange数据库编号分别为JPST001240和PXD027118(https://repository.jpostdb.org/preview/87715052764a54fd4732c6;访问密钥:9786)。

### 1.8 数据库检索

将得到的质谱文件加载到Spectronaut软件^[25]^中,使用不依赖于谱图库的检索模式(directDIA),所使用的数据库为人源蛋白质数据库(包含20168个条目,www.uniprot.org)。所使用的酶设置为“Trypsin”,最大漏切数设置为2,固定修饰设定为半胱氨酸(C)上的氨基甲酰甲基化(carbamidomethyl),可变修饰设置为丝氨酸(S)/苏氨酸(T)/酪氨酸(Y)的磷酸化、甲硫氨酸(M)的氧化以及蛋白质N端的乙酰化;其他参数保持为Spectronaut软件的默认参数,即质量偏差和保留时间偏差都设置为“Dynamic”,校正因子(correlation factor)设置为1。前体离子和蛋白质鉴定的假阳性率(false discovery rate, FDR)均设置为1%。在排除掉干扰肽段后,选择丰度最高的3条肽段进行蛋白质的定量,得到的蛋白质定量结果使用“global normalization”策略进行矫正,鉴定到的磷酸化位点筛选条件为“PTM Site Probability”,得分≥0.75。数据库检索产生的磷酸化蛋白质组鉴定和定量结果以及生物信息学富集分析结果已上传至Science Data Bank(https://cstr.cn/31253.11.sciencedb.09392)。

## 2 结果与讨论

### 2.1 高剂量E2诱导HeLa细胞死亡的表型分析

很多临床研究和实验数据表明,高剂量(μmol/L~mmol/L)E2可以诱导多种肿瘤细胞死亡,促进氧化应激,产生与低剂量(pmol/L~nmol/L)E2截然相反的效应,但相关内在调控过程及作用机制尚不明确^[7⇓⇓-10]^。Welshons等^[7]^系统考察了10^-14^~10^-4^ mol/LE2对MCF7细胞增殖的影响,发现10^-14^~10^-11^ mol/L的E2能够促进MCF7细胞增殖,但当剂量增加到10^-6^mol/L以上时,E2转而抑制MCF7细胞增殖。近期,王晓东课题组^[3]^在研究卵巢衰老的过程中发现,E2浓度为2.5~10 μmol/L时可以诱导HeLa细胞凋亡,其半抑制浓度为1.5 μmol/L。为了验证这一结果,我们首先采用CCK-8实验和基于台盼蓝染色的细胞计数实验以及显微镜拍照,探究了μmol/L水平的E2对HeLa细胞增殖能力、活力水平及形态的影响。

#### 2.1.1 高剂量E2抑制HeLa细胞增殖

为了探究高剂量E2对HeLa细胞增殖能力的影响,首先利用CCK-8实验分别对1~10 μmol/L E2处理1、2天后的HeLa细胞数目进行分析,每种处理进行6个复孔分析。结果如图1所示,与文献报道一致,除了1 μmol/L E2处理1天的细胞样品外,E2对HeLa细胞增殖表现出了显著的抑制作用(显著性水平(*p*)<0.01)。与DMSO处理组相比,随着E2处理时间的延长及暴露剂量的增加,孔板内HeLa活细胞数逐渐减少;其中,5 μmol/L E2分别处理HeLa细胞1、2天后,相对于DMSO处理组,分别抑制了32.9%(中值)和74.1%(中值)的HeLa细胞增殖,表明在1~10 μmol/L剂量条件下,E2对HeLa细胞的增殖抑制效应以剂量依赖和时间依赖的形式逐渐增强。

**图 1:**
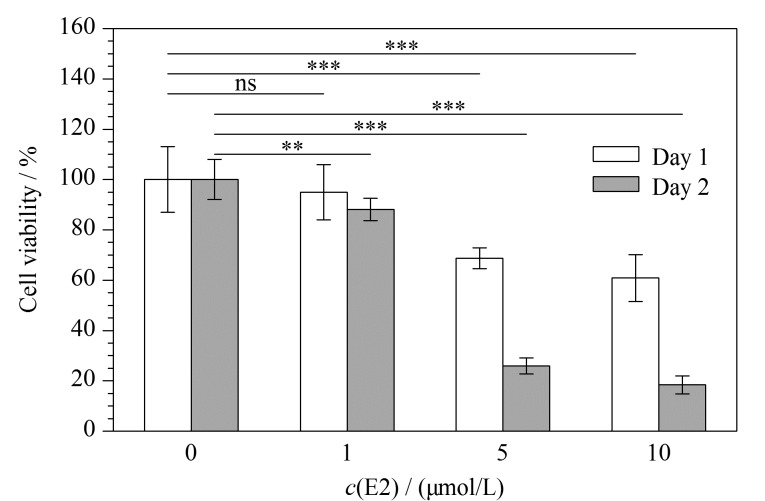
高剂量E2对HeLa细胞活率的影响(*n*=6)

#### 2.1.2 高剂量E2诱导HeLa细胞死亡

为了进一步探究高剂量E2对HeLa细胞活力的影响,首先利用显微镜拍照分析了5 μmol/L E2或DMSO处理不同时间后的HeLa细胞形态。结果如图2所示,在低倍镜(10×)下,5 μmol/L E2处理1天后的HeLa细胞开始皱缩变圆,部分细胞质膜塌陷,边缘模糊;处理2~3天后的HeLa细胞皱缩更为明显,聚结成团并悬浮,处于贴壁状态的细胞边缘变得粗糙,细胞内容物外溢;同时,DMSO处理的HeLa细胞随着处理时间的延长细胞数量逐渐增加,细胞形态完整,以上结果说明高剂量E2可以诱导HeLa细胞死亡。

**图 2:**
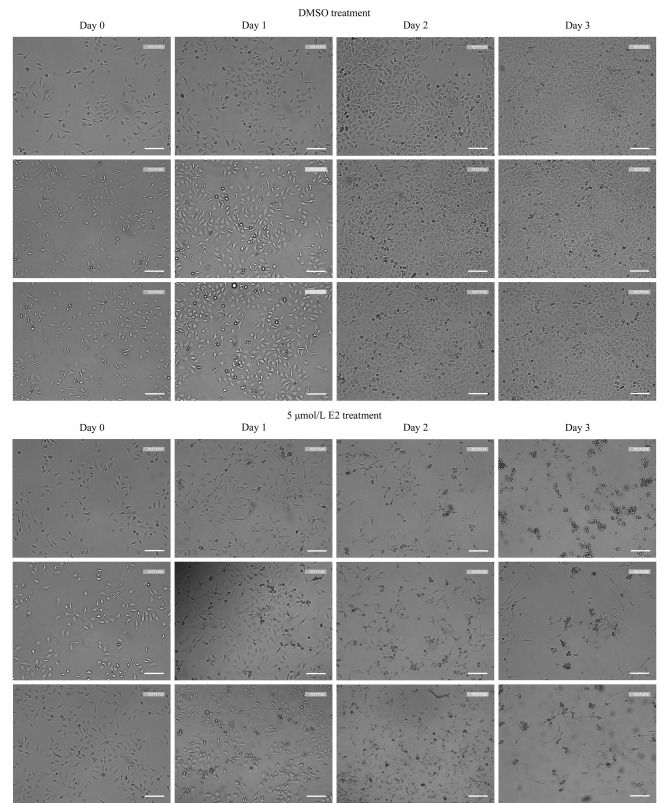
高剂量(5 μmol/L)E2对HeLa细胞形态的影响

利用台盼蓝染色对上述不同处理条件下的活细胞和死细胞数目及比例进行统计分析。结果如图3a和3b所示,与空白对照组相比,用5 μmol/L E2处理1天后,活细胞的数目发生了显著下调(数目中值由4.2×10^5^变化到3.2×10^5^, *p*=0.0045),但活细胞的比例未发生显著变化(数目中值由86.3%变化到82.3%, *p*=0.2499);用5 μmol/L E2处理2天后,活细胞的数目和比例均发生了显著下调(数目中值由6.7×10^5^变化到2.0×10^5^, *p*=0.0002;比例中值由77.0%变为到49.7%, *p*=0.0194);用5 μmol/L E2处理3天后,活细胞的数目和比例进一步发生了显著下调(数目中值为8.2×10^5^变化到0.8×10^5^, *p*=0.0002;比例中值由79.0%变化到16.3%, *p*=0.0194)。此外,DMSO处理组细胞数量逐渐增加,活细胞的比例未发生显著变化(*p*<0.05)。以上结果与文献报道一致,说明高剂量E2可以诱导HeLa细胞死亡^[3]^。

**图 3:**
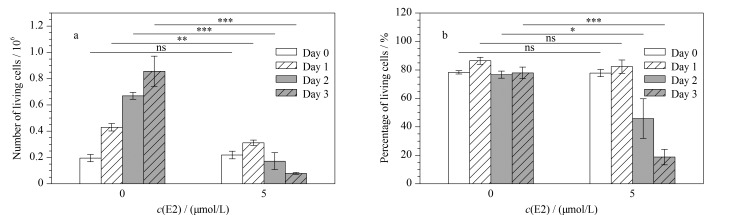
高剂量(5 μmol/L)E2对HeLa(a)活细胞数和(b)活细胞比例的影响(*n*=3)

### 2.2 高剂量E2致死效应的内在调控过程解析

#### 2.2.1 Ti^4+^-IMAC结合DIA定量技术用于磷酸化位点的高灵敏鉴定

基于以上表型分析结果,将5 μmol/L E2或DMSO处理两天后的HeLa细胞样品依次进行细胞破碎、蛋白质提取、还原烷基化反应和滤膜辅助的蛋白酶解,并将得到的酶解肽段进行磷酸化肽段富集,除盐后进行LC-MS/MS分析(图4a)。基于固相萃取(SPE)模式的Ti^4+^-IMAC磷酸肽富集策略具有操作自动化、样品损失少和无样品污染等优点^[22]^,因此,本工作中将其用于E2和DMSO处理后的HeLa细胞样品中磷酸化位点的深覆盖鉴定。结果如图4b所示,结合DIA定量技术,从各样品中分别定量到发生在2406~2602个磷酸化蛋白质上的6701~7829个磷酸化位点(PTM site probability≥0.75),最终,从HeLa细胞中共定量到2874个蛋白质上的10092个磷酸化位点。进一步分析发现,E2和DMSO处理组各样品间磷酸化位点定量结果的皮尔森相关性系数(Pearson correlation coefficient)中值分别为0.935和0.933(图4c),变异系数(coefficient of variation)中值分别为0.196和0.204(图4d),说明磷酸肽富集操作的平行性及DIA定量结果的重现性均较好。

**图 4:**
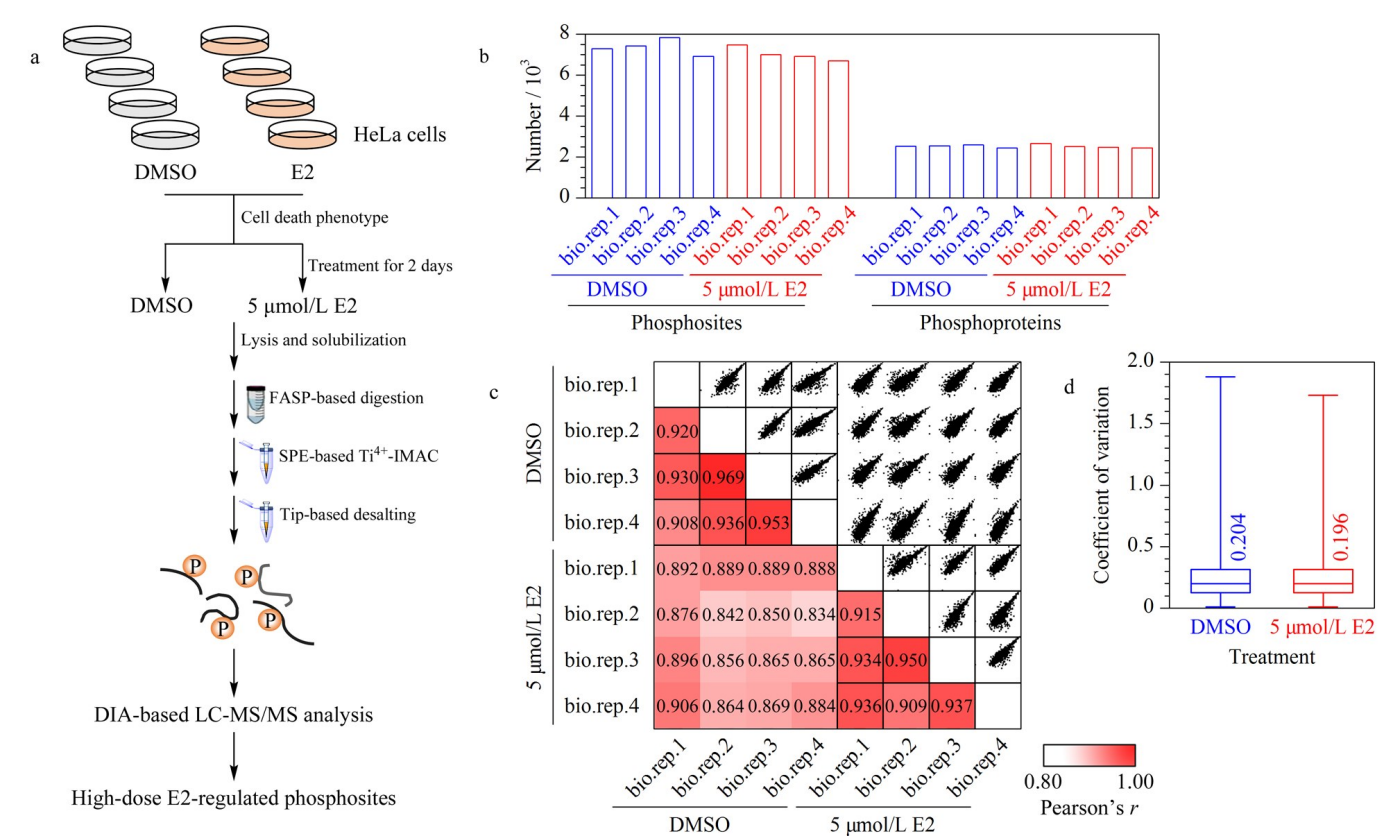
Ti^4+^-IMAC结合DIA定量技术用于磷酸化位点深覆盖鉴定

#### 2.2.2 高剂量E2调控磷酸化位点的定量筛选

为了全面解析参与调控E2致死效应过程的磷酸化位点,通过Perseus软件^[26]^对E2和DMSO处理组之间磷酸化位点修饰水平的倍数变化(fold change, FC)及其显著性水平进行分析。首先,将定量结果中满足E2和(或)DMSO处理组中至少3个样品不存在缺失值或满足E2和DMSO处理组均有两个样品不存在缺失值的9154个磷酸化位点进行后续分析。对于这些磷酸化位点,选择“total matrix”模式进行插值(imputation)以处理定量结果中的缺失值,选择“Benjamini-Hochberg-based independent”模式进行*t*检验分析(FDR<5%)。结果如图5所示,与DMSO处理组相比,这些磷酸化位点中约有5.9%(537个)和2.8%(387个)磷酸化位点的修饰水平在5 μmol/L E2处理后分别发生了显著上调或下调(|log_2_(FC)|≥1, *p*<0.05)。E2处理显著改变了599个蛋白上的924个磷酸化位点的丰度水平(|log_2_(FC)|≥1, *p*<0.05),说明这924个磷酸化位点可能参与调控E2致死效应过程。此外,在磷酸化蛋白质组定量结果中,有453个磷酸化位点(对应325个蛋白质)单独发生在E2或DMSO处理组(一组中至少3次质谱分析不含缺失值,另一组中均为缺失值),这些磷酸化位点可能在E2处理后发生了磷酸化或去磷酸化事件,进而参与E2致死效应调控过程。综上所述,Ti^4+^-IMAC结合DIA定量技术共发现741个蛋白质上的1218个磷酸化位点可能参与调控HeLa细胞内的E2致死效应过程,这些信息可以为后续E2作用机制研究以及疾病的治疗提供一些数据参考。

**图 5:**
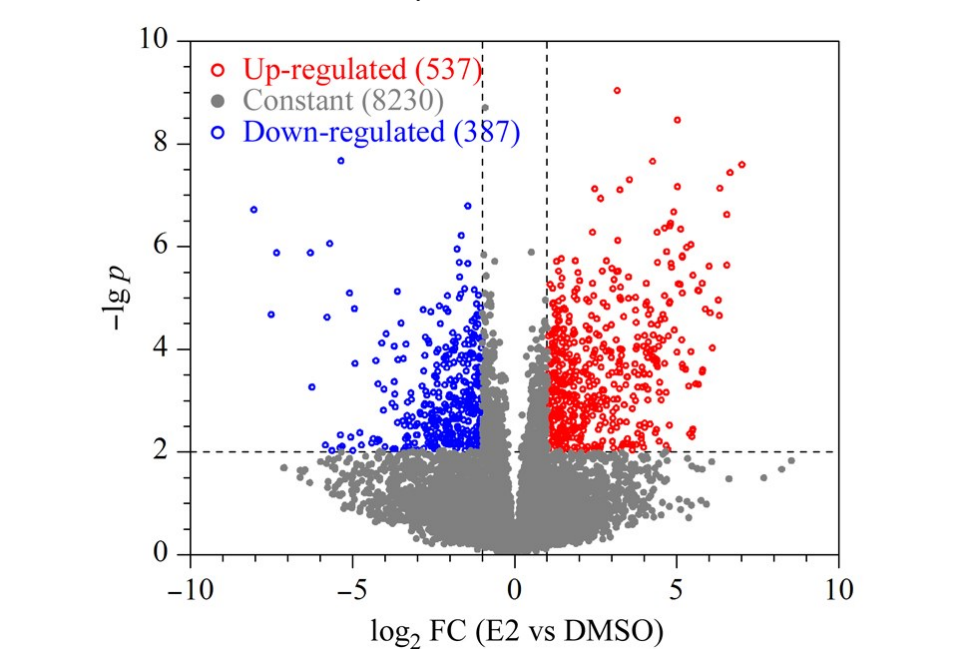
高剂量E2调控磷酸化位点的定量筛选

#### 2.2.3 E2诱导细胞死亡的内在调控过程解析

作为一种瞬时可逆的翻译后修饰,蛋白质磷酸化几乎在所有的细胞生命活动(包括细胞生长、有丝分裂、信号传导和细胞周期控制)中都发挥着至关重要的调控作用^[27]^。本文利用现有蛋白质功能注释数据库(包括Gene Ontology(GO)、Kyoto Encyclopedia of Genes and Genomes(KEGG) pathway等)对上述筛选的含有差异磷酸化位点的蛋白质进行标注,进而初步探究高剂量E2致死效应的内在调控过程。将筛选到的参与高剂量E2调控过程的599和325个磷酸化蛋白质在DAVID(database for annotation, visualization and integrated discovery)软件^[28]^中进行GO和KEGG通路富集分析(图6a~d)。将人源蛋白质组作为背景蛋白质库,并同时将磷酸化蛋白质组定量结果在GESA(Gene Set Enrichment Analysis)软件^[29]^上进行KEGG和WikiPathway分析(图6e)。

**图 6:**
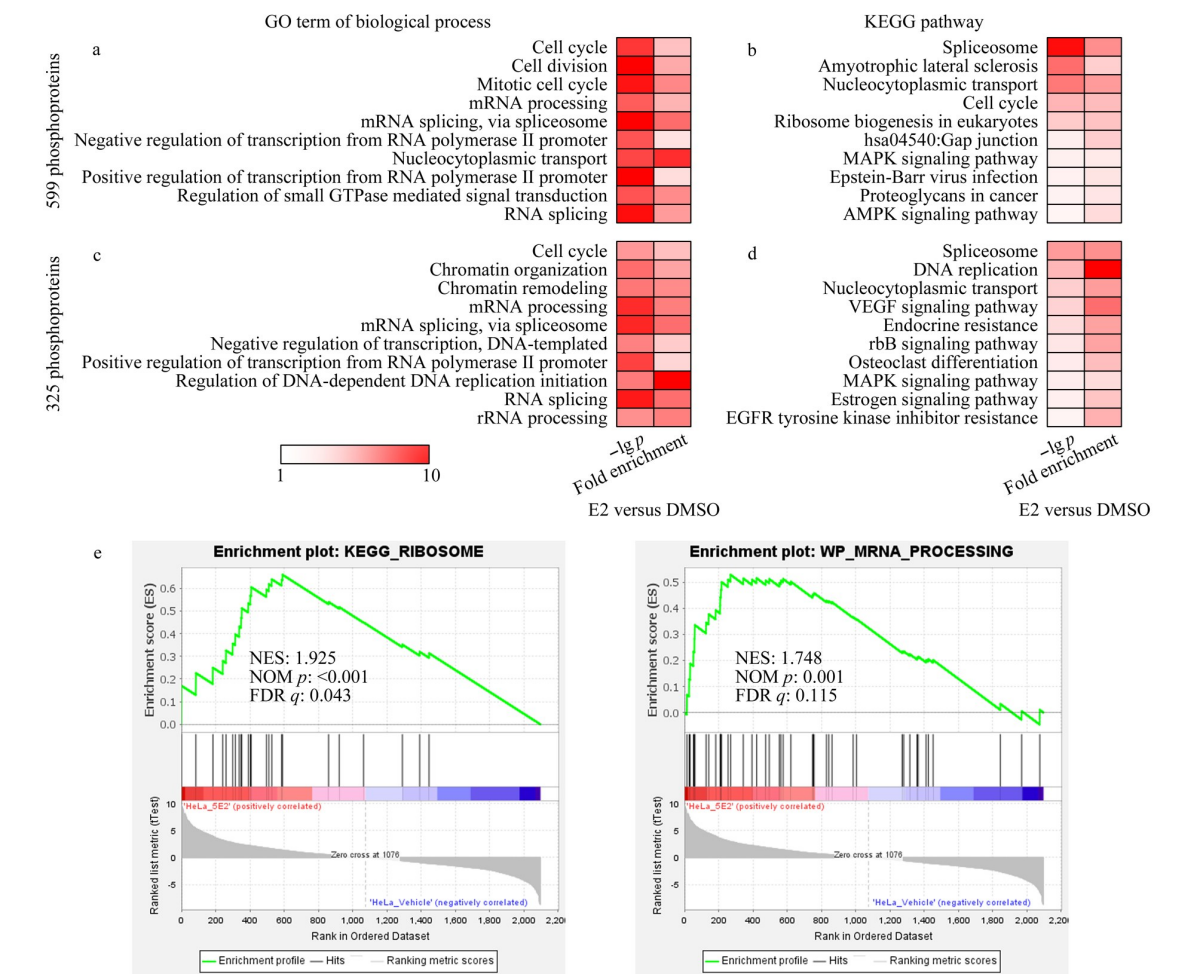
高剂量E2调控磷酸化蛋白质的生物信息学分析

与细胞表型分析结果一致(图1~3),以上两种方式筛选的两组E2调控的磷酸化蛋白质显著(*p*<0.00005)富集了参与细胞周期调控的蛋白质,这进一步验证了高剂量E2可抑制HeLa细胞增殖。此外,信使核糖核酸(mRNA)加工/剪接、核质转运、转录等细胞过程中的相关蛋白质也被显著(*p*<0.01)富集在这两组磷酸化蛋白质中(图6a、6c)。举例来说,高剂量E2处理可显著(*p*<0.05)上调多个核糖体相关的磷酸化位点,包括RPL17(60S ribosomal protein L17)的S5和RPS3(40S ribosomal protein S3)的T221(图7)。GESA富集分析结果也显示,核糖体及mRNA加工通路相关蛋白显著(*p*<0.01)富集于高剂量E2上调的磷酸化蛋白质中(图6e)。之前的报道中^[3]^, E2-PDE3A-SLFN12复合物通过结合核糖体上的RNA来抑制蛋白质转录,诱导细胞死亡。结合本实验的磷酸化蛋白质组定量结果,推测高剂量(μmol/L)E2可通过结合核糖体上的RNA,干扰mRNA加工/剪切,抑制核糖体的蛋白质翻译(包括一些核孔蛋白),导致蛋白质核质运输功能受损,促进HeLa细胞发生凋亡。同时,599个磷酸化蛋白质富集到的信号通路主要涉及剪接体、细胞周期、核质转运、核糖体生物合成、丝裂原活化蛋白激酶(MAPK)信号通路等;325个磷酸化蛋白质富集到的信号通路主要涉及剪接体、DNA复制、核质转运、MAPK信号通路、内分泌抵抗、雌激素信号通路以及表皮生长因子受体(EGFR)信号通路(图6b、6d)。很多剪接体通路相关的磷酸化位点,包括ACIN1(apoptotic chromatin condensation inducer in the nucleus)、HNRNPM(heterogeneous nuclear ribonucleoprotein M)、HNRNPA1(heterogeneous nuclear ribonucleoprotein A1)及SRSF2(serine/arginine-rich splicing factor 2),在E2致死效应过程中发生了明显上调,进一步说明高剂量(μmol/L)E2可能影响了mRNA的剪切/加工过程。值得注意的是,在高剂量(μmol/L)E2处理后,ACIN1蛋白中S240、S243及S328位点上的磷酸化修饰水平发生了显著上调(*p*<0.05),而在S710和S714位点上的磷酸化修饰水平发生了显著下调(*p*<0.05)。据文献[30]报道,在凋亡过程中ACIN1蛋白质参与诱导染色质聚结,这些位点可能在高剂量(μmol/L)E2诱导HeLa细胞凋亡过程中发挥着重要调节作用。

**图 7:**
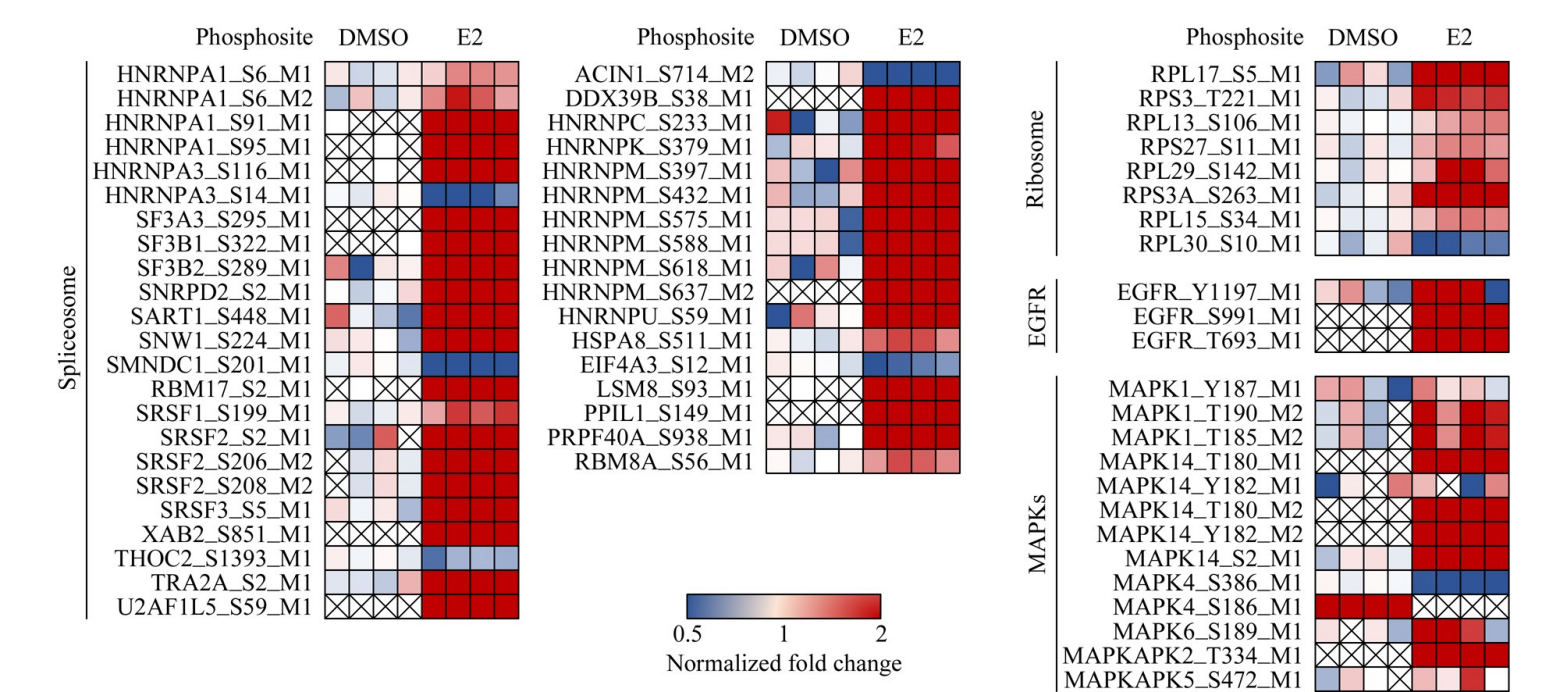
E2调控磷酸化位点的定量信息

此外,在高剂量E2处理后,EGFR上多个磷酸化位点的修饰水平发生了明显变化。其中,T693在E2处理后发生了显著上调(*p*<0.05),而这一磷酸化行为也参与表皮生长因子(EGF)、诺考达唑(nocodazole)等分子调控的细胞生长过程(phosphoSitePlus数据库, https://www.phosphosite.org/homeAction.action)。并且,在高剂量E2处理后,参与调控EGFR蛋白质T693位点磷酸化事件的激酶MAPK1(ERK2)蛋白质T190和T185位点也发生了磷酸化(图7),表明MAPK1-EGFR可能在高剂量E2诱导HeLa细胞死亡的过程中发挥着重要的调节作用。MAPK信号通路参与调控细胞生长、发育、分化、凋亡等多种细胞生理活动。除了MAPK1外,在所得的磷酸化蛋白质组数据中,MAPK14的T180、Y182、S2位点和MAPKAPK2(MAP kinase-activated protein kinase 2)的T334位点的磷酸化修饰水平均发生了显著上调(*p*<0.05)。据文献[31]报道,MAPK14和MAPKAPK2在滋养层细胞分化过程中发挥重要调节作用,其中MAPKAPK2可以通过激活MAPK14来调控细胞过程。这些磷酸化位点很可能通过调控雌激素诱导的细胞凋亡进而参与人胎盘侵染和发育过程的调节。此外,MAPK4参与调控细胞周期,其S386和S186位点的磷酸化修饰水平在高剂量E2处理后均发生了显著下调(*p*<0.05)。综上,我们推测EGFR和MAPK信号通路可能参与高剂量E2致死效应过程的调控,促进HeLa细胞发生凋亡,并可能为后续人胚胎流产的治疗提供功能性磷酸化位点信息。

## 3 结论

本工作聚焦高剂量E2致死效应这一表型,将基于固相萃取模式的Ti^4+^-IMAC磷酸肽富集方法结合基于DIA的蛋白质组定量技术,成功筛选了可能参与调控高剂量E2致死过程的741个蛋白质上的1218个磷酸化位点,这些蛋白质除了参与细胞周期的调控外,还主要富集在核糖体、mRNA剪接/加工、核质转运及转录等细胞过程;同时发现,EGFR和MAPK信号通路可能参与调控HeLa细胞的E2致死效应过程。所得到的磷酸化蛋白质组定量数据可以为后续高剂量E2致死效应研究提供数据支持。
